# Sciatica

**Published:** 1889-06

**Authors:** B. Simmons

**Affiliations:** 30 Alberto Terrace, Sydney, New South Wales


### SCIATICA.

Editors of the Homœopathic Physician:

I am collecting the characteristic symptoms of remedies useful in sciatica; may I beg your readers to forward me any information they can on the treatment of this disease, so as to make this attempt the more practically useful to those who individualize their cases. Only characteristic symptoms are asked for. The *Materia Medica* will contain all that refers to this malady that is published in Hering's *Condensed*, Lippe's, Cowperthwaite's, and Guernsey's *Materia Medicas;* then will follow a repertory of the same, with a few concomitants. There is, perhaps, no disease which so thoroughly tests the skill of the homœopathic physician as the selection of the simillimum in cases of sciatica, and it may be hoped that the publication of the above in your valuable pages may help the younger members of the profession to win more laurels for the homœopathic healing art. Old-school physic can do very little for sciatica, but the true simillimum, judiciously given, will act as promptly in this as in other intractable maladies,

I am, sir, yours faithfully,

B. SIMMONS, M. D.

30 ALBERTO TERRACE, SYDNEY,
NEW SOUTH WALES, April 12th, 1889.

